# 9-Methyl-β-carboline inhibits monoamine oxidase activity and stimulates the expression of neurotrophic factors by astrocytes

**DOI:** 10.1007/s00702-020-02189-9

**Published:** 2020-04-13

**Authors:** Sebastian Keller, Witold Henryk Polanski, Christoph Enzensperger, Heinz Reichmann, Andreas Hermann, Gabriele Gille

**Affiliations:** 1 Department of Neurology, Technische Universität Dresden, Fetscherstr. 74, 01307 Dresden, Germany; 2 Department of Neurosurgery, Technische Universität Dresden, Fetscherstr. 74, 01307 Dresden, Germany; 3 Institute of Pharmacy, Friedrich Schiller University of Jena, Philosophenweg 14, 07743 Jena, Germany; 4 SmartDyeLivery GmbH, Botzstraße 5, 07743 Jena, Germany; 5 Translational Neurodegeneration Section “Albrecht-Kossel”, Department of Neurology and Center for Transdisciplinary Neurosciences Rostock (CTNR), University Medical Center Rostock, University of Rostock, 18147 Rostock, Germany; 6 German Center for Neurodegenerative Diseases (DZNE) Rostock/Greifswald, 18147 Rostock, Germany

**Keywords:** Dopaminergic neurons, Astrocytes, 9-Methyl-β-carboline, Inhibition of monoamine oxidase A and B, Neurotrophic factors

## Abstract

β-Carbolines (BC) are pyridoindoles, which can be found in various exogenous and endogenous sources. Recent studies revealed neurostimulative, neuroprotective, neuroregenerative and anti-inflammatory effects of 9-methyl-BC (9-Me-BC). Additionally, 9-me-BC increased neurite outgrowth of dopaminergic neurons independent of dopamine uptake into these neurons. In this study, the role of astrocytes in neurostimulative, neuroregenerative and neuroprotective properties of 9-me-BC was further explored.

9-Me-BC exerted anti-proliferative effects without toxic properties in dopaminergic midbrain and cortical astrocyte cultures. The organic cation transporter (OCT) but not the dopamine transporter seem to mediate at least part the effect of 9-me-BC on astrocytes. Remarkably, 9-me-BC stimulated the gene expression of several important neurotrophic factors for dopaminergic neurons like Artn, Bdnf, Egln1, Tgfb2 and Ncam1. These factors are well known to stimulate neurite outgrowth and to show neuroprotective and neuroregenerative properties to dopaminergic neurons against various toxins. Further, we show that effect of 9-me-BC is mediated through phosphatidylinositol 3-kinase (PI3K) pathway. Additionally, 9-me-BC showed inhibitory properties to monoamine oxidase (MAO) activity with an IC50 value of 1 µM for MAO-A and of 15.5 µM for MAO-B. The inhibition of MAO by 9-me-BC might contribute to the observed increased dopamine content and anti-apoptotic properties in cell culture after 9-me-BC treatment in recent studies. Thus, 9-me-BC have a plethora of beneficial effects on dopaminergic neurons warranting its exploration as a new multimodal anti-parkinsonian medication.

**Electronic supplementary material:**

The online version of this article (10.1007/s00702-020-02189-9) contains supplementary material, which is available to authorized users.

## Introduction

β-Carbolines (BC) are pyridoindoles, which can be synthesized from tryptophan or tryptophan-like indolamines and can be found in numerous exogenous and endogenous sources (Piechowska et al. 2019). They structurally resemble the Parkinson’s disease (PD) causing substance 1-methyl-4-phenyl-1,2,3,6-tetrahydropyridine (MPTP) and showed neurotoxic effects on dopaminergic neurons, especially as methylated forms (e.g. 2,9-dimethyl-β-carbolinium ion [2,9-dime-BC +]) (Hamann et al. 2006; Neafsey et al. 1995; Cruz-Hernandez et al. 2018; Sammi et al. 2018). Inhibition of the complex I of the respiratory chain, induction of apoptosis and activation of microglia are the main compounds of this toxic effects of 2,9-dime-BC + (Ostergren et al. 2006; Polanski et al. 2010; Hamann et al. 2006; Hans et al. 2005). Actually, elevated concentrations of BCs (2,9-dime-BC + , harman and norharman) were detected in the cerebrospinal fluid of PD patients (Matsubara et al. 1995; Kuhn et al. 1996; Louis et al. 2014). As the second most frequent neurodegenerative disorder after Alzheimer’s disease, PD is arising from multifactorial pathomechanisms. Mitochondrial dysfunction with inhibition of the respiratory chain, increased production of reactive oxygen species, disturbance of the proteasome, lysosomal dysfunction, gliosis and neuroinflammatory processes are believed to play a major role (Jenner and Olanow 2006; Lee et al. 2019; Schapira et al. 2016). Furthermore, PD is characterized by the formation of protein aggregates called Lewy bodies that mainly consist of α-synuclein (Burre et al. 2018). S-phase kinase-associated protein 1A (Skp1) plays also a key role in ubiquitin-dependent protein depletion (Bai et al. 1996) and was found to be decreased in the substantia nigra of PD patients (Grunblatt et al. 2004). Also, zink finger proteins (ZFP) seem to play a crucial role in misfolding of proteins (Brahmachari et al. 2019). The physiological functions of α-synuclein are still insufficiently known (Ghosh et al. 2017). α-synuclein plays an important role in cell survival (Jensen et al. 2003; Perez et al. 2002; Drolet et al. 2006) and synaptic metabolism (Ben et al. 2009). On the one hand, an overexpression of α-synuclein leads to cell death via inflammation (Theodore et al. 2008), inhibition of chaperone activity (Hinault et al. 2010), transcriptional dysfunction (Yuan et al. 2010) and lysosomal dysfunction (Moors et al. 2016); on the other hand, there is an ongoing dispute on the role of α-synuclein in the degenerative process of dopaminergic neurons. Substances that reduce the propensity of α- synuclein aggregation under conditions of intracellular oxidative stress might be helpful for treatment of PD (Shen et al. 2019).

Up to now, no curative treatment of PD has been found and there is an ongoing investigation for substances which exert protective effects on dopaminergic neurons or are able to delay their degeneration. During the past years, promising advances have been made in this direction with deprenyl, rasagiline, (−)-epigallocatechin-3-gallate and iron chelators, for example (LeWitt 1991; Mandel et al. 2004, 2007; Zhu et al. 2007). Surprisingly, recent studies revealed that 9-methyl-β-carboline (9-me-BC) has emerged as a promising agent for treatment of PD. In contrast to other neurotoxic BCs, 9-me-BC showed neuroprotective effects on dopaminergic neurons against 2,9-dime-BC + toxicity and neuroregenerative properties after chronic rotenone treatment in in vitro experiments in murine primary cells (Polanski et al. 2010). Furthermore, in vivo studies revealed also neuroregenerative properties against MPP + treatment in rats (Wernicke et al. 2010). Also, anti-inflammatory properties were observed by attenuating the proliferation of activated microglia and decreasing the gene expression of various inflammatory cytokines and receptors (Polanski et al. 2010). Furthermore, 9-me-BC lowered the protein levels of α-synuclein (Polanski et al. 2010), a hallmark of PD. Additionally, 9-me-BC stimulated the number of tyrosine hydroxylase immunoreactive (TH +) neurons by increasing the protein and gene expression of TH in pre-existing dopa decarboxylase immunoreactive (DDCir) neurons and inducing the gene expression of TH-relevant transcription factors like gata binding protein 2 (Gata2), gata binding protein 3 (Gata3), cAMP response element-binding protein (Creb) and CREB-binding protein (Crebbp) (Polanski et al. 2010). Hypoxia-inducible factor 1 (HIF-1) and its encoded gene Egl nine homolog 1 (Egln1) are also known to induce TH expression (Nguyen et al. 2007) and have potential as a medical target for PD (Zhang et al. 2011). Finally, 9-me-BC increased the expression of neurotrophic factors like glial cell line-derived neurotrophic factor (GDNF) and brain-derived neurotrophic factor (BDNF) in in vivo studies (Wernicke et al. 2010) and stimulated neurite outgrowth of dopaminergic neurons (Polanski et al. 2010; Beck 1994). Neurotrophin 3 (NT-3) also stimulates TH + neurons and dopamine uptake in mesencephalic neuronal cultures (Hyman et al. 1994). Additionally, protective properties of NT-3 and BDNF to dopaminergic neurons against axotomization were reported (Hagg 1998). The neural cell adhesion molecule1 (NCAM1) is potentially involved in 9-me-BC-induced neuritogenesis since inhibition of protein kinases A and C, which are involved in the signaling pathway of NCAM, blocked the stimulatory effects of 9-me-BC (Polanski et al. 2010). A comprehensive review has been published that illustrates the signaling pathways to be important in NCAM-mediated neurite outgrowth (Ditlevsen et al. 2008). Moreover, NCAM and GDNF were shown to cooperate in promoting the neurite outgrowth of 6-OHDA injured dopaminergic neurons, while GDNF alone was sufficient to restore TH expression in these cells (Cao et al. 2008). Actually, promising clinical trials with Neurturin (NRTN) (Ceregen Inc, clinical trial) are implemented, while clinical trials with GDNF failed to show benefits in PD treatment (Kordower et al. 1999; Lang et al. 2006; Whone et al. 2019) and produced side effects (Nutt et al. 2003). Further clinical trials were started with cerebral dopamine neurotrophic factor [CDNF; also known as Arginine rich, mutated in early-stage tumors (armet)] for treatment of PD (Herantis Pharma Plc, clinical trial). Preclinical studies showed comparable neuroprotective properties like for GDNF (Lindholm et al. 2007).

Transforming growth factor-beta 2 (TGF-β2) and TGF-β3 are important regulators of neuronal survival although they are probably not neurotrophic by themselves, but can increase the potency and neuroprotective action of other neurotrophic factors like GDNF or fibroplastic growth factor (FGF-2) (Krieglstein et al. 2002). Therefore, TGF-βs are also essential for the development and survival of midbrain dopaminergic neurons during embryogenesis (Farkas et al. 2003; Reyes-Corona et al. 2017). Artemin (Artn), neurturin (Nrtn) and persephin (Pspn) as part of the GDNF family have been shown to promote the number and morphological differentiation of cultured ventral mesencephalic dopaminergic neurons (Zihlmann et al. 2005). Artn acts through the PI3K/Akt pathway (Omodaka et al. 2014) and improves regeneration of injured neurons (Wong et al. 2015). A recent study showed that Pspn has neuroprotective effects in animal models of PD (Yin et al. 2015).

Leucine-rich repeat kinase 2 (LRRK2) is a protein, whose mutations cause a familial form of PD. LRRK2 seems to play a role in the maintenance of neuronal process length and complexity (MacLeod et al. 2006) and regulates protein translation. In contrast, LRRK2 mutations can deregulate protein translation resulting in age-dependent loss of dopaminergic neurons (Imai et al. 2008). Recently, an essential and unexpected role of LRRK2 in the regulation of protein homeostasis during aging was shown in mice implying that LRRK2 mutations cause PD and cell death via impairment of protein degradation pathways, leading to α-synuclein accumulation and aggregation over time (Tong et al. 2010).When the dopamine transporter (DAT), by which 9-me-BC is completely taken up into dopaminergic neurons (Hamann et al. 2008), was blocked, the stimulatory effects on the number of dopaminergic neurons were abolished but a further increase of the neurite outgrowth was observed (Polanski et al. 2010). This discrepancy led to the perception that the effect on neurite outgrowth is independent of the uptake of 9-me-BC into dopaminergic neurons but perhaps contingent on uptake into astrocytes by an organic cation transporter (OCT).

In the last years, astrocytes were researched preferentially because of their important role in synapse formation, function and plasticity and homeostasis of neurotransmitters was elucidated. They are also well known to produce a variety of factors, which stimulate and promote neurite outgrowth and exert neuroprotective properties. Further, there is evidence that astrocytes play a direct role in neurodegenerative diseases like PD (Rappold and Tieu 2010; Booth et al. 2017). In this study, the role of astrocytes in neurostimulative, neuroregenerative and neuroprotective properties of 9-me-BC was further explored. To this end, we used primary dopaminergic mesencephalic cell cultures, astrocyte-depleted cultures and cortical astrocyte cultures. Further, effects on proliferation of astrocytes and possible toxic effects of 9-me-BC were elucidated. Gene expression analyses of neurotrophic factors in cortical astrocyte cultures were performed to outline the important role in 9-me-BC-mediated effects. Finally, possible inhibitory properties to monoamine oxidase of 9-me-BC were investigated.

## Materials and methods

### Materials

C57BL/6 mice were delivered by Charles River Wiga GmbH (Sulzfeld, Germany). Four-well culture dishes, Nunclon™ Flasks and white F96 MicroWell™ Plates were obtained from NUNC (Wiesbaden, Germany). Cutasept F was from BODE Chemie (Hamburg, Germany). Norharman was purchased from Acros Organics (Fisher Scientific, Schwerte, Germany). Anti-tyrosine hydroxylase (MAB5280) was from Millipore Corporation (Bedford, MA, USA). DNase I, Penicillin–Streptomycin and Cell Proliferation ELISA BrdU (colorimetric) were purchased from Roche (Mannheim, Germany). Dulbecco’s modified Eagle’s medium (DMEM), Dulbecco’s phosphate-buffered saline (DPBS), Hank’s balanced salt solution, B-27 supplement (without antioxidants Propidium iodide, Hoechst 33342, Trypsin (1×) and Superscript VILO cDNA synthesis kit were purchased from Invitrogen (Karlsruhe, Germany). The Vectastain ABC Elite kit (Universal), and the Vector VIP (purple) were obtained from Vector Laboratories (Burlingame, USA). Poly-d-lysine, Triton X-100, glucose, fetal calf serum, Histochoice, dimethyl sulfoxide (DMSO), human monoamine oxidase B, clorgyline, pargyline and Hepes buffer were from Sigma-Aldrich (Taufkirchen, Germany). MAO-Glo™ Assay was from Promega (Mannheim, Germany). The RNAprotect Cell Reagent, RNeasy Mini Kit, RNase-Free DNase Set and QiaShredder were delivered by Qiagen (Hilden, Germany). Primers and RT2 qPCR Master Mix were purchased from SABiosciences (Frederick, MD, USA). Disprocynium 24 (D24) was purchased from Servier (Suresnes cedex, France). CellTiter-Blue® Cell Viability Assay was obtained from Promega (Mannheim, Germany). ToxiLight BioAssay Kit was delivered from Lonza (Cologne, Germany). LY294002 hydrochlorid (2-[4-Morpholinyl]-8-phenyl-4H-1-benzopyran-4-one-hydrochloride) and sulpiride ((*RS*)-*N*-[(1-Ethylpyrrolidin-2-yl)-methyl]-2-methoxy-5-sulfamoylbenzamid) were from TOCRIS biosciences (Bristol, UK). F4/80 antibody was from AbD Serotec (Duesseldorf, Germany).

### Synthesis of 9-methyl-β-carboline

Synthesis of 9-me-BC was performed as described by (Hamann et al. 2008).

### Animals

Pregnant mice (C57BL/6, between 3 and 6 months old) were cared and handled in accordance with the guidelines of the European Union Council (86/609/EU) for the use of laboratory animals.

### Dopaminergic cell culture

Animals were killed on gestation day 14 by asphyxiation. Dopaminergic cell cultures were prepared as described earlier (Polanski et al. 2010). Half of the basic medium (composition see (Hamann et al. 2008)) was changed on the first day in vitro (DIV) and on the 3rd DIV, two-thirds of the basic medium were changed. On the 5th DIV, half of the basic medium was replaced with serum-free Dulbecco’s modified Eagle’s medium (DMEM) containing 2% B-27. Serum-free supplemented DMEM medium was used for feeding from the 6th DIV and was subsequently replaced every second day.

### Astrocyte-depleted culture

Astrocyte-depleted cultures were prepared identically to dopaminergic cultures but from the first DIV, cells were treated with serum-free supplemented DMEM medium. Subsequently, the medium was replaced every second day.

### Cortical astrocyte culture

Animals were killed at birth by decapitation. The heads were disinfected with Cutasept F (BODE Chemie, Hamburg, Germany), brains were dissected under a stereoscope (Zeiss, Stemi DV4) and the cortices of the hemispheres were abraded. Afterwards, the cortices were transferred into DPBS (Dulbecco’s phosphate-buffered saline) containing Petri dishes. The meninges were deducted and the cortices were briefly cut into small pieces in a small volume of DPBS. 2 mL of trypsin solution and 100 µL of DNase I solution were added and the tissue was subsequently incubated in a water bath at 37 °C for 7 min. This reaction was stopped by adding 2 mL of basic medium. Then, the tissue was centrifuged at 800 rpm for 3 min and the supernatant was discarded. The tissue pellet was incubated with 3-mL basic medium and 100-µL DNAse I and triturated with a fire-polished Pasteur pipette. Dissociated cells were plated in Nunclon™ Flasks from NUNC (Wiesbaden, Germany) in basic medium. Half of the medium was changed on the 1st DIV, on the 3rd DIV 2/3 of the medium was changed. On the 5th DIV, half of the medium was changed and on the 6th and on the 8th DIV, the complete medium was replaced. For further cultivation, on the 8th DIV before changing medium, the cultures were constantly shaken for 8 h at 37 °C to remove non-astrocytic cells. On the 10th DIV, cells were incubated with trypsin solution for 10 min and after adding basic medium centrifuged at 800 rpm for 3 min. The supernatant was discarded and the tissue pellet was incubated with basic medium. The astrocytes were plated in 4-well culture dishes at a density of 50,000 cells/cm^2^ in basic medium. Afterwards, medium was completely changed every second day. Co-staining with GFAP, F4/80 and Hoechst revealed less than 1% non-astrocytic cells within the culture.

### Immunocytochemical staining of tyrosine hydroxylase immunoreactive neurons

Cultures were rinsed carefully with DPBS (pH 7.2) and fixed with Histochoice Tissue Fixative (SigmaAldrich, Taufkirchen, Germany) for 30 min at room temperature (22 °C). Cells were permeabilized with 0.4% Triton X-100 for 15 min at 22 °C and incubated with anti-TH antibody (final dilution 1: 3500) overnight at 4 °C. Then, cultures were treated with the biotinylated secondary antibody for 1 h [Vectastain ABC Elite kit universal from Vector Laboratories (Burlingame, CA, USA)] and finally, the avidin–biotin–horseradish peroxidase complex (Vectastain ABC Elite kit universal) was added for 1 h at 22 °C. The reaction product was developed with Vector VIP (purple). Cells were counted with a Zeiss inverted microscope (Zeiss Ax overt 35) in 21 fields (1.302 mm^2^/field) in each well at 100 × magnification. Images were taken with a computer-driven digital camera (Leica DC350 FX, Wentzler, Germany) on an inverted microscope (Leica DM IRE2 HC FLUO).

### Treatment with Disprocynium 24

Disprocynium 24 (D24, Servier, Suresnes cedex, France) is an inhibitor of the organic cation transporters 1, 2 and 3 (OCT1, OCT2, OCT3). A stock solution of D24 was prepared in water and further diluted in basic medium. Because of the bad solubility, the stock solution was incubated in an ultrasonic bath at 30 °C. Incubation with D24 started 30 min before treatment with 9-me-BC for 48 h at the 12th DIV.

### Treatment with LY294002 hydrochloride and sulpiride

For inhibition of the phosphatidylinositol 3-kinase (PI3K) pathway, a stock solution of the LY294002 hydrochlorid (2-[4-Morpholinyl]-8-phenyl-4*H*-1-benzopyran-4-one hydrochloride), for blocking the dopamine receptors 2 and 3, a stock solution of sulpiride ([S]-[−]-5-Aminosulfonyl-*N*-[(1-ethyl-2pyrrolidinyl)methyl]-2-methoxybenzamide) from TOCRIS biosciences (Bristol, UK) in dimethyl sulfoxide (DMSO) were prepared and further diluted in basic medium. As final concentrations, cells were incubated with 10-µM LY294002 hydrochloride or 20-µM sulpiride 30 min before adding 9-me-BC for 48 h at the 10th DIV.

### Measurement of the adenylate kinase activity

The adenylate kinase activity as a marker of cell injury was measured with the ToxiLight BioAssay Kit from Lonza (Cologne, Germany) according to the manufacturer’s instructions. 20 µL of the supernatant (on 14th DIV) per well was used for the luminescence measurement with a microplate reader (TECAN GENios, 1-s integration time, 100-ms and 150-ms exposure time; Crailsheim, Germany).

### Measurement of lactate dehydrogenase release

The release of lactate dehydrogenase (LDH) into the medium as a marker of cell injury was measured spectrophotometrically at 490 nm with a reference at 600 nm with the LDH Cytotoxicity Detection Kit (Roche) according to the manufacturer’s instructions (briefly described in Hamann et al. 2008).

### Staining of necrotic/apoptotic and total nuclei

Stock solutions of Hoechst 33342 (10 mg/mL in H_2_O) and PI (1 mg/mL in H_2_O) were prepared with final concentrations of PI (0.65 mg/mL) and of Hoechst 33342 (7.5 mg/mL). Cultures were incubated with each substance for 5 min, washed and observed in serum-free supplemented DMEM medium. Apoptotic/necrotic cells were detected by the uptake of PI into cell nuclei, since vital cells did not incorporate PI. In contrast, Hoechst 33342 was incorporated into nuclei of all cells. Differentiation of necrosis or apoptosis was not possible with this method. This ratio demonstrates the ratio of dying cells to all cells and was declared in percent. Images were taken at 100 × magnification with a computer-driven digital camera (Leica DC350 FX,Wetzlar, Germany) on an inverted microscope (Leica DM IRE2 HC FLUO, Wentzler, Germany) equipped with an incubator and temperature control for live cell experiments. Red fluorescence (PI) was visualized with the TRITC filter [excitation 515–560 Band Pass (BP)/emission 590 Long Pass] and blue fluorescence (Hoechst 33342) with the DAPI filter [excitation 340–380 Band Pass (BP)/emission 425 Long Pass]. LEICA FW4000-I was used as software. Image analysis was performed densitometrically with ADOBE PHOTOSHOP (Munich, Germany).

### Measurement of the viability of astrocytes

For measurement of the viability of astrocytes the CellTiter-Blue^®^ Cell Viability Assay from Promega (Mannheim, Germany) was used according to the manufacturer’s instructions. On the 14th DIV, cultures were incubated with 150-µL CellTiter Blue^®^ per well for 4 h at 37 °C. Absorbance was measured at 600 nm, and reference at 570 nm with a microplate reader (TECAN GENios, Crailsheim, Germany).

### Bromdesoxyuridine (BrdU)-ELISA

For quantification of cell proliferation, incorporation of BrdU was measured with the Cell Proliferation ELISA BrdU (colorimetric) from Roche (Mannheim, Germany). Therefore, 75-µL BrdU labeling solution was added to each well and incubated for 6 h at 37 °C. Afterwards, the supernatant was discarded and the cells were treated with 300 µL FixDenat per well for 30 min at 22 °C. Then, the cultures were incubated with 300-µL Anti-BrdU Peroxidase working solution for 60 min and washed three times with PBS. Finally, cells were treated with 300-µL substrate solution for 10 min at 22 °C and the reaction was stopped by adding 75-µL H2SO4. After 5 min, absorbance was measured at 450 nm and reference at 690 nm with a microplate reader (TECAN GENios, Crailsheim, Germany).

### Measurement of the monoamine oxidase (MAO) activity

Human MAO-A and -B activity was determined with the MAO-Glo™ Assay from Promega (Mannheim, Germany). Since this assay did not contain MAO-B, this enzyme was purchased from Sigma-Aldrich (Taufkirchen, Germany). Therefore, a stock solution of 9-me-BC in MAO reaction buffer [100-mM HEPES buffer (pH 7.5), 5% glycerol] was prepared. For the measurement of the MAO-B activity, additionally 10% DMSO was added to this buffer. Further steps were performed according to the manufacturer’s instructions. The incubation time of the MAO reaction was 1 h; the luciferase reaction was stopped after 20 min. For the luminescence measurement, white F96 MicroWell™ Plates from NUNC (Wiesbaden, Germany) and a microplate reader (TECAN GENios, Crailsheim, Germany) were used. The activity of MAO-A and -B was 120 µU/µL. For blanc value, the measurement was performed without MAO-A or -B. As reference inhibitors, clorgyline for MAO-A and pargyline for MAO-B from Sigma-Aldrich (Wiesbaden, Germany) were used.

### Reverse transcription quantitative real-time PCR

For reverse transcription, the Superscript VILO cDNA synthesis kit (Invitrogen) was used according to the manufacturer’s instructions. cDNA was stored for at most 6 days at – 80 °C. RTqPCR was performed with the Mx3000P system from Stratagene. Tubes (Cat.no.: 401428) and plates (Cat.no.: 410088) were also purchased from Stratagene. Primers and RT2 qPCR Master Mix were from SABiosciences and used as stated in the manufacturer’s instructions. Primers (Cat.no. in brackets) for actin beta (Actb; PPM02945A), Arginine rich, mutated in early-stage tumors (armet; PPM27631A), artemin (Artn; PPM04308B), brain-derived neurotrophic factor (Bdnf; PPM03006B), epidermal growth factor (Egf; PPM03707C), epidermal growth factor receptor (Egfr, PPM03714F), EGL nine homolog 1 (Egln1; PPM31485A), fibroblast growth factor 2 (Fgf2; PPM03040B), fibroblast growth factor receptor 2 (Fgf2r; PPM 03706E), glial cell line-derived neurotrophic factor (Gdnf; PPM04315E), hypoxia-inducible factor 1, alpha subunit (Hif1a; PPM03799B), Leucine-rich repeat kinase 2 (Lrrk2; PPM28453A), Neural cell adhesion molecule 1 (Ncam1; PPM03672F), nerve growth factor (Ngf; PPM03596B), Neurturin (Nrtn; PPM04326E), neurotrophin 3 (Ntf3; PPM04325A), Persephin (Pspn; PPM04335A), S-phase kinase-associated protein 1A (Skp1a; PPM02914A), Transforming growth factor, beta 1 (Tgfb1; PPM02991A), Transforming growth factor, beta 2 (Tgfb2; PPM02992A), Transforming growth factor, beta 3 (Tgfb3; PPM02993A), Zinc finger protein 91 (Zfp91; PPM03003E) were used. The thermal profile was run with 1 cycle at 95 °C for 10 min and with 40 cycles each at 95 °C for 15 s and subsequently at 60 °C for 1 min. Data analysis was performed with MxPro–Mx3000P v4.01 software from Stratagene. Changes in gene expression were calculated with the ΔΔCt method. Actin beta was used as a reference gene. Statistics were performed with normalized Δ*C*_t_ values (normalized to control).

### Measurement of toxicity and cell viability

To investigate if 9-me-BC exert toxic effects on cortical astrocytes (Fig. suppl.), cells were treated with various concentrations of 9-me-BC (10 µM, 30 µM, 50 µM, 90 µM and 150 µM) for 48 h (14–16 DIV) and the toxic potential was detected with the ToxiLight BioAssay Kit (Lonza, Cologne, Germany), which measures the adenylate kinase concentration in the supernatant. The viability was measured with CellTiter-Blue^®^ Cell Viability Assay from Promega (Mannheim, Germany).

### Statistics

Every experiment was run in quadruplicate (four wells in each experiment) and was repeated at least three times. Results are presented as mean values ± SEM. Data were tested for normality using the Shapiro–Wilk test. Statistical significance was calculated using the one-way analysis of variance (ANOVA), Kruskal–Wallis (*H*) test or Mann–Whitney *U* test. The ANOVA and the Kruskal–Wallis test were followed by a Bonferroni test.

## Results

### 9-me-BC increases the number of dopaminergic neurons

**Fig. 1:**
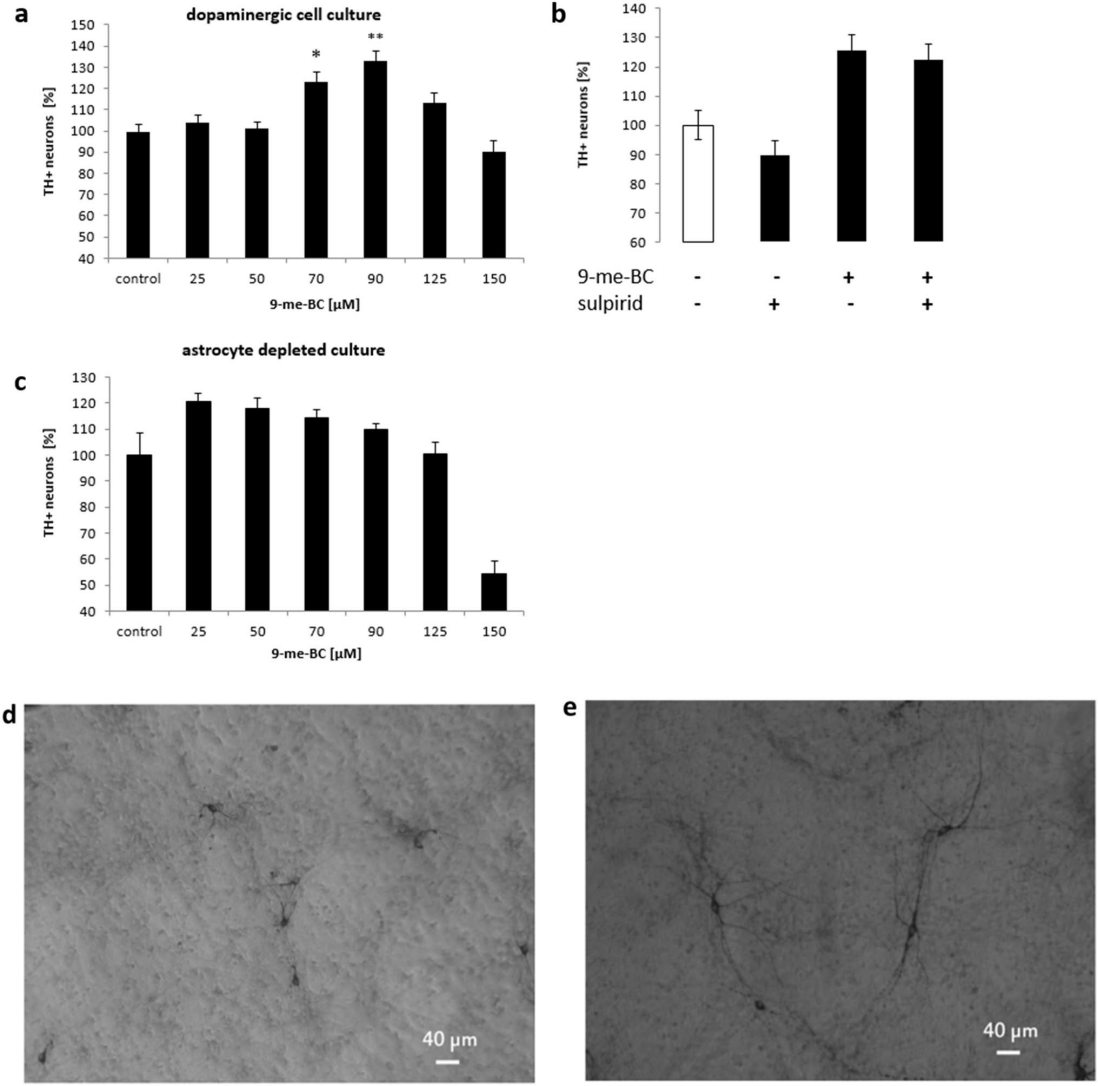
Concentration-dependent increase of tyrosine hydroxylase immunoreactive (TH +) neurons in dopaminergic cell culture (**a**) and astrocyte-depleted culture (**c**) after treatment with 9-methyl-β-carboline (9-me-BC) for 48 h [10–12 days in vitro (DIV)]. **b** Impact of co-treatment with sulpiride and 9-me-BC (20 µM, added 30 min before 9-me-BC treatment) on the stimulatory effects of 9-me-BC (90 µM) to TH + neurons. Cells were incubated with both substances for 48 h (10–12 DIV). Differences to control values were calculated with the Kruskal–Wallis (*H*) test followed by a Bonferroni test, *p < 0.05, **p < 0.001. Data represent the mean ± SEM of three independent experiments in quadruplicate. **d**, **e** Stained tyrosine hydroxylase immunoreactive neurons in an astrocyte-depleted culture (100 × magnification). Control at 12 DIV (**d**) and culture after incubation with treatment with 9-me-BC (50 µM) for 48 h (10–12 DIV) (**e**)

When mesencephalic dopaminergic cultures were treated with 9-me-BC for 48 h (10–12 DIV), an increased number of TH + neurons in a concentration-dependent manner was observed (Fig. 1a). This stimulatory effect reached its maximum with 33 ± 8% of additional TH + neurons after 90 µM 9-me-BC treatment. To elucidate whether this effect is mediated by dopamine receptors 2 and 3, cultures were co-treated with sulpiride and 9-me-BC. Sulpiride is clinically used as a neuroleptic drug and can cause extrapyramidal side effects. Incubation with 20-µM sulpiride for 48 h (10–12 DIV) alone not significantly decreased the number of TH + neurons. Treatment with 70-µM 9-me-BC increased the number of TH + neurons by 27 ± 7% which was not influenced by co-treatment with 20-µM sulpiride, which indicates that the stimulatory effects of 9-me-BC are dopamine receptor independent (Fig. 1b).

We next asked whether astrocytes do influence survival of TH + neurons. For this, astrocyte growth was suppressed by serum deprivation (named as astrocyte-depleted culture). We did observe a similar effect of 9-me-BC treatment with the tendency to increase TH + neurons (not significant) (Fig. 1c). Obviously, lower concentrations of 9-me-BC (25 µM) were necessary to exert a maximal increase of TH + neurons compared to mesencephalic dopaminergic cultures (90 µM). Treatment of astrocyte-depleted cultures with 150-µM 9-me-BC decreased the number of TH + neurons by 50%. Comparatively, the same concentration of 9-me-BC in mesencephalic dopaminergic cultures showed only a slight reduction of dopaminergic neurons. Furthermore, an impaired morphology of TH + neurons was observed in astrocyte-depleted cultures (Fig. 1d), which was considerably improved after treatment with 9-me-BC (Fig. 1e).

### Anti-proliferative properties of 9-me-BC in cortical astrocyte cultures without toxic effects

**Fig. 2:**
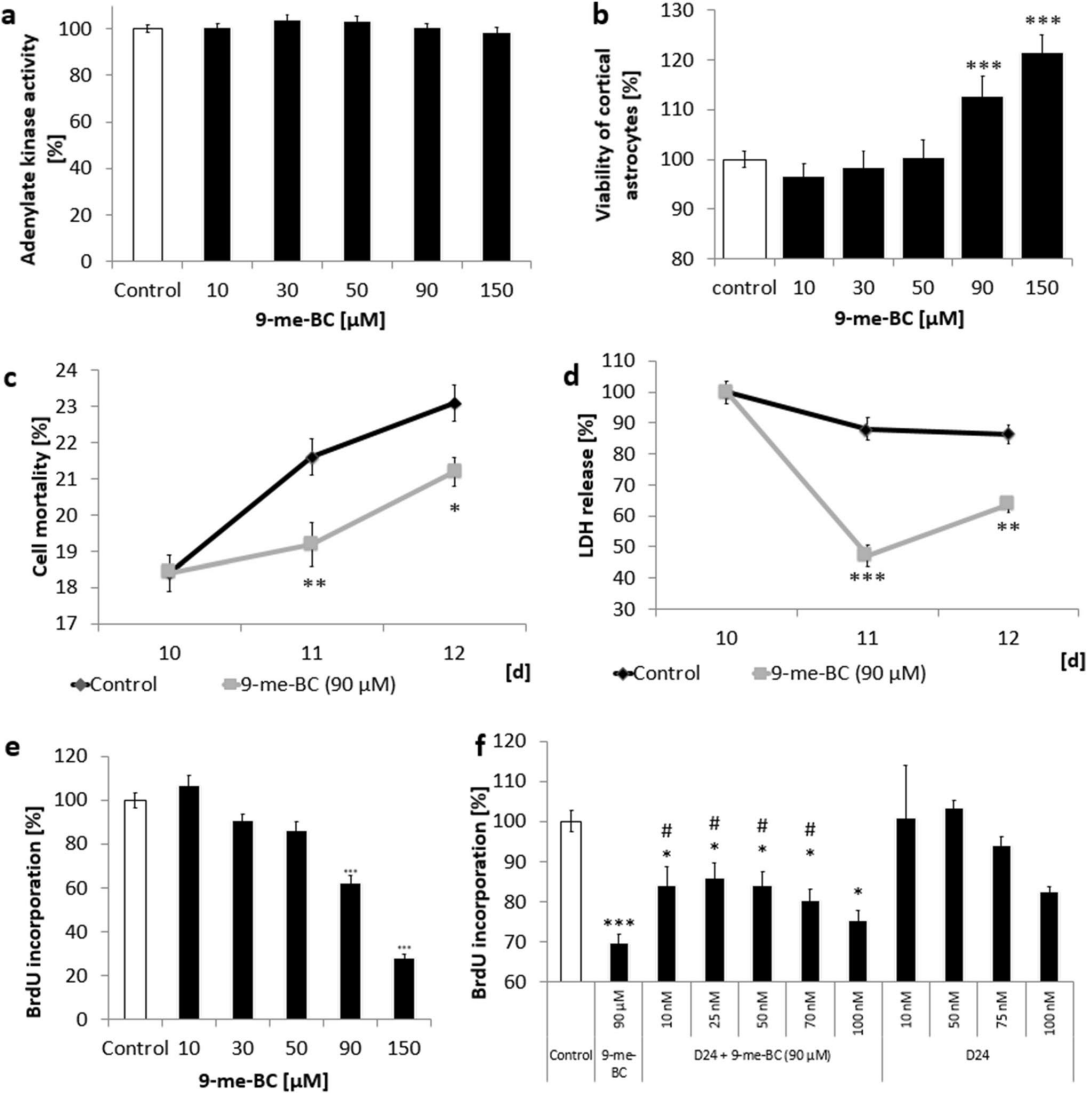
Effects on the adenylate kinase activity (**a**, **b**) and to the viability of cortical astrocytes after 9-methyl-β-carboline (9-me-BC) treatment for 48 h [12–14 days in vitro (DIV)]. Cell mortality (**c**) and lactate dehydrogenase (LDH) release (**d**) on 10–12 DIV, when dopaminergic cultures were treated with 90 µM 9-me-BC for 48 h (10–12 DIV). **e** Concentration-dependent effects of 9-me-BC (90 µM) treatment for 48 h (12–14 DIV) to the BrdU incorporation of cortical astrocytes. **f** Effects of co-treatment with 9-me-BCn (90 µM) and with various concentrations of dysprocynium (D24) for 48 h (12–14 DIV) to the BrdU incorporation of cortical astrocytes. Data represent the mean ± SEM of 3 (**b** **c**, **d**, **f**), 4 (**e**) and 5 (**a**) independent experiments in quadruplicate. Differences were compared to respective control values (*) or 90 µmolar 9-me-BC (#) and were calculated with the Kruskal–Wallis (*H*) test followed by a Bonferroni test; *p < 0.05, **p < 0.001, ***p < 0.0001, #*p* < 0.05, ##*p* < 0.001

9-meBC did not increase the adenylate kinase concentration with any applied concentration, implicating that 9-me-BC shows no toxic effects on cortical astrocytes (Fig. 2a).

Astrocyte cultures showed an increased viability by 12% after 90-µM 9-me-BC incubation and 20% after 150-µM 9-me-BC treatment was observed (Fig. 2b). These results and confirming studies with MTT (data not shown) and measurement of the proportion of PI- and Hoechst 33342 incorporation demonstrate that 9-me-BC shows no toxic effects on astrocytes in administrated concentration.

When astrocyte cultures were incubated with 9-me-BC, no increase of dead cells was measured but in contrast, the further increase of dead cells, which was observed in control cultures, was even lowered significantly from 23% in control cultures to 21% in treated cultures with 9-me-BC (Fig. 2c). Coincidentally, the LDH release on the 12 DIV was significantly decreased to 73% compared with the control culture (Fig. 2d).

Since the decrease of the dead cells could also be explained by a forced proliferation of astrocytes, a BrdU-ELISA in cortical astrocyte cultures was performed. Therefore, cells were treated with 9-me-BC in various concentrations for 48 h (12–14 DIV). Lower concentrations of 9-me-BC (10 µM, 30 µM and 50 µM) showed no significant changes in BrdU incorporation but 90-µM and 150-µM 9-me-BC reduced significantly (*p* < 0.0001) the amount of BrdU incorporation by 39 ± 4% and 71 ± 6% in a concentration-dependent manner (Fig. 2e). This indicates an antiproliferative effect of 9-me-BC to cortical astrocytes.

Until now, detailed uptake mechanisms of 9-me-BC into astrocytes are unknown. To elucidate whether 9-me-BC mediates its effect on astrocytes by the OCT, cultures were treated with the OCT inhibitor dysprocynium 24 (D24) and anti-proliferative properties of 9-me-BC were measured. When cultures were co-treated with 90-µM 9-me-BC and the OCT inhibition dysprocynium 24 (D24), the anti-proliferative effects of 9-me-BC were inhibited significantly in a concentration-dependent manner (Fig. 2g). Considering that 90-µM 9-me-BC treatment reduced the BrdU incorporation by 31% and co-treatment with 50-nM D24 resulted in 17% inhibition of BrdU incorporation, the OCT seems to play an important but not exclusive role in mediating the effect of 9-me-BC on astrocytes. It is not known if the OCT was completely blocked or if another till-now-unknown transporter is also responsible for 9-me-BC uptake, since higher D24 concentrations also reduced the proliferation of astrocytes by itself. Further studies are necessary to show detailed uptake kinetics of 9-me-BC into astrocytes.

### Increased gene expression of several neurotrophic factors after 9-me-BC treatment

**Fig. 3:**
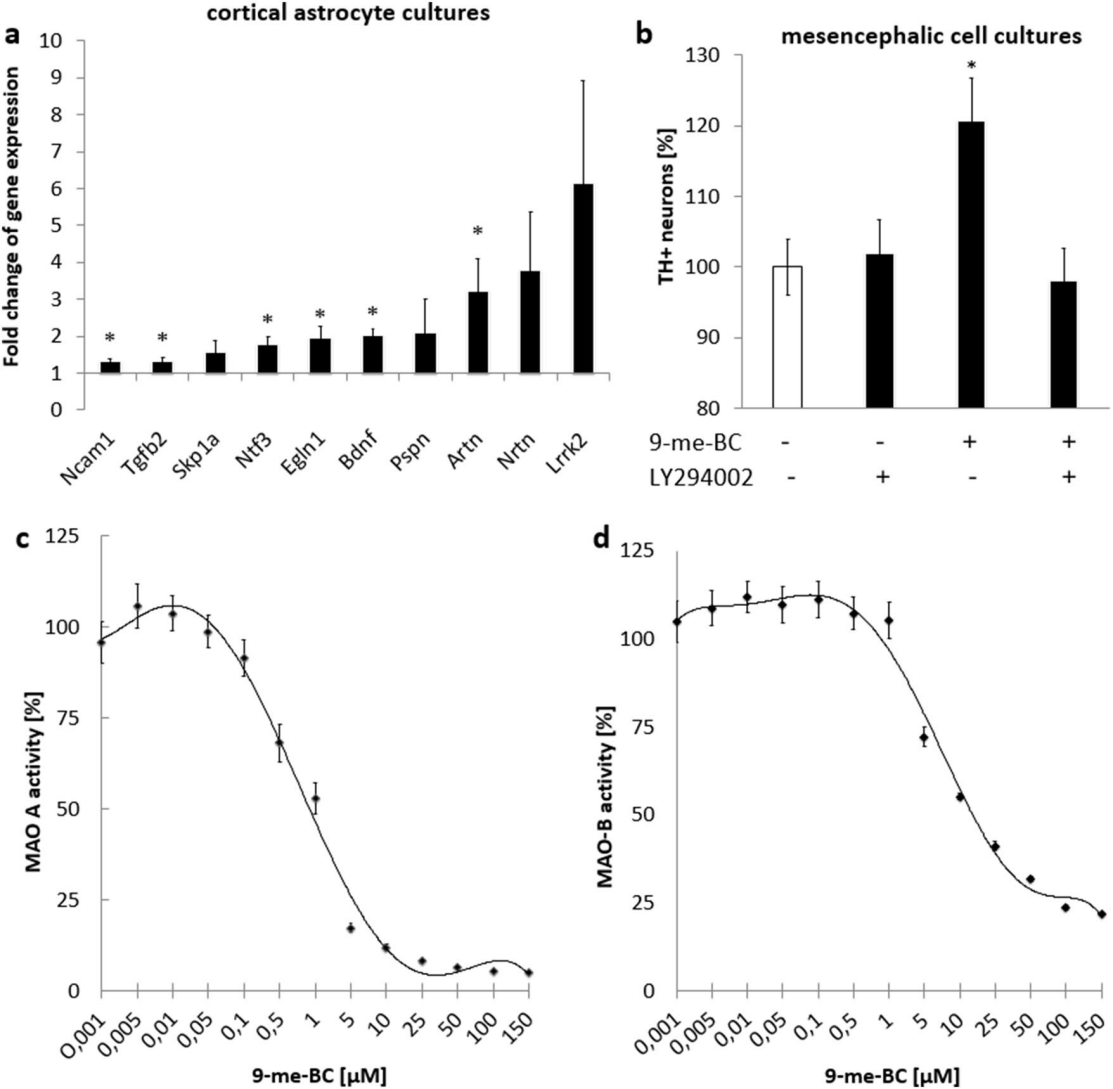
**a** Gene expression of cortical astrocytes after treatment with 9-methyl-β-carboline (9-me-BC; 90 µM) for 48 h [12–14 days in vitro (DIV)] compared to untreated cultures. Gene expression was analyzed with RT-qPCR and beta actin was used as a reference gene. **b** Affection of co-treatment with LY294002 (10 µM, added 30 min before 9-me-BC treatment) on the stimulatory effects of 9-me-BC (90 µM) to tyrosine hydroxylase immunoreactive (TH +) neurons in mesencephalic cell cultures. Cells were incubated with both substances for 48 h (10–12 DIV). MAO-A (**c**) and -B (**d**) inhibition. Statistical differences were calculated with the Mann–Whitney *U* test with normalized Δ*C*_t_ values (normalized to control), *p < 0.05 compared to control at 14 DIV. Data are expressed as mean ± SEM of three (**a**, **c**, **d**) and four (**b**) independent experiments in duplicate

To reveal if astrocytes play an important role in the stimulatory effect of 9-me-BC, gene expression of several neurotrophic factors in cortical astrocyte cultures was investigated. After 48 h of treatment with 90-µM 9-me-BC (12–14 DIV), the gene expression of the GDNF family ligands artemin (Artn) was significantly increased by 3.2-fold (*p* < 0.01). The expression of neurturin (Nrtn) and persephin (Pspn) showed no significant increase. Furthermore, gene expression of Tgf-β2 but not Tgf-β1 was slightly but significantly up-regulated by 1.4-fold in cortical astrocytes after 9-me-BC treatment. Gene expression of Bdnf and Ncam1 were also significantly elevated by twofold and 1.4-fold. Actually, the gene expression of Ntf3 was increased significantly by 1.8-fold after 9-me-BC treatment. Gene expression of LRRK2 did not change significantly after 9-me-BC treatment. 9-me-BC increased also the gene expression of Skp1 by 1.5-fold significantly, which might lead to an increased turnover of proteins, especially α-synuclein (Fig. 3a).

Treatment with the LY 294002, an inhibitor of the Pi3K/Akt pathway, for 48 h (10–12 DIV, 10 µM), blocked the stimulatory effects of 90-µM 9-me-BC to TH + neurons completely, implicating that the PI3K/Akt pathway seems to play a central role in neurostimulative effects on dopaminergic neurons by 9-meBC (Fig. 3b).

### Inhibition of MAO-A and -B by 9-me-BC

Human MAO-A and -B activity was determined with the MAO-Glo™ Assay from Promega. 9-me-BC showed IC50 values of 1 µM for MAO-A inhibition (Fig. 3c) and of 15.5 µM for MAO-B inhibition (Fig. 3d).

## Discussion

Although especially methylated, quaternized BCs (e.g., 2,9-dime-BC +) are suspected to cause PD since they show neurotoxic effects on dopaminergic neurons (Hamann et al. 2006; Neafsey et al. 1995), 9-me-BC exerted unexpectedly neurostimulative, neuroprotective, neuroregenerative and anti-inflammatory properties (Hamann et al. 2008; Polanski et al. 2010). The previously described stimulatory effects on dopaminergic neurons were confirmed in this study and furthermore, there is strong evidence that astrocytes play a crucial role in stimulatory effects and neurite outgrowth, induced by 9-me-BC. The astrocytes seem to determine the optimal concentration of 9-me-BC for stimulatory properties and affect the morphology of neurons. Recent studies showed that 9-me-BC has to enter the neurons by the dopamine transporter (DAT) to exert its neurostimulative effects (Hamann et al. 2008; Polanski et al. 2010). Since there were no significant neurostimulative effects of 9-me-BC in astrocyte-depleted cultures and the stimulation of the neurite outgrowth was further observed although the uptake into dopaminergic neurons was blocked (Polanski et al. 2010), astrocytes may play an important role in the effects exerted by 9-me-BC. The seemingly toxic effects of 9-me-BC in astrocyte-depleted culture might be based on the lack of antitoxic shielding of astrocytes accompanied by an overdose of 9-me-BC, since astrocytes are able to shield neurons from potential toxic effects of various substances (Vibulsreth et al. 1987; Watts et al. 2005). It is known that astrocytes play a crucial role in adaptive immune response in the brain and are able to regulate neuroinflammatory pathways (Colombo and Farina 2016; Tremblay et al. 2019). Since 9-me-BC showed anti-inflammatory properties (Polanski et al. 2010), further studies will show whether these effects are also mediated through astrocytes or are a result of direct interaction with microglia.

Several studies have previously shown that 9-me-BC increased the gene expression of numerous neurotrophic factors like Gdnf, Bdnf, Cdnf, Cbln1, Ngf, Cntf, Gdnf receptor α and β, sonic hedgehog (Shh), wingless-type mouse mammary tumor virus (MMTV) and integration site family, member 1 and member 5a and of important transcription factors for dopaminergic neurons [engrailed homeobox 1, nuclear receptor related 1 (Nurr1) and paired-like homeodomain transcription factor 3 (Pitx3), Gata2, Gata3, Creb and Crebbp] (Polanski et al. 2010; Wernicke et al. 2010; Hamann et al. 2008). The neurotrophic effects of 9-me-BC might be explainable by the consistent expression of neurotrophic factors by astrocytes. Such factors like artemin (Artn), neurturin (Nrtn) and persephin (Pspn) were upregulated after 9-me-BC treatment.

TGF-β2, Bdnf, NCAM1 and Ntf3 were also upregulated after 9-me-BC treatment and, thus, 9-me-BC was able to improve the morphology of TH + neurons even in astrocytes-depleted cultures. This improvement seems to be dependent on the PI3K/Akt pathway, which plays an important role in neurite outgrowth and neuronal cell survival (Neiiendam et al. 2004) and is shared by a range of neurotrophic factors and not due to an increased proliferation of astrocytes. In this study, a not selective PI3K inhibitor was used to screen whether the PI3K/Act pathway may play a role in 9-me-BC-dependent effects. Since the used inhibitor also binds to mechanistic Target of Rapamycin (mTOR) and glycogen synthase kinase 3 (GSK3) (Yang et al. 2019), that are both downstream targets of the PI3K/Act pathway (Liu et al. 2009), the exact mechanism remains unclear. Further research with more selective inhibitors is needed to elucidate the exact downstream mechanism. On the contrary, 9-me-BC showed even anti-proliferative properties in cortical astrocyte cultures without being toxic. In this context, there is evidence that the organic cation transporter (OCT) seems to play an important but not exclusive role in the uptake mechanisms of 9-me-BC into astrocytes, since OCT subtypes are also responsible for dopamine uptake (Duan and Wang 2010) and for uptake of MPP + (Shang et al. 2003). There are three main OCT subtypes (OCT1, OCT2 and OCT3), which occur also in the brain. Recent studies revealed that OCT1 and OCT2 are involved in MPTP induced toxicity to dopaminergic neurons (Lin et al. 2010). Further, OCT3 immunoreactive astrocytes were detected, which showed protective properties against MPTP toxicity when they were inhibited (Cui et al. 2009). The OCT inhibitor DC24 was able to rescue 9-me-BC effects until a concentration up to 100 nM; however, this effect was becoming smaller in higher DC24 concentrations. Indeed, higher DC24 concentrations showed (yet insignificant) trend to inhibit cell proliferation themselves and were even toxic in higher concentrations (data not shown). Since there are reports that D24 may even stimulate OCT in rats in concentrations below 1 µM (Amphoux et al. 2010), we can not prove how the D24 acted in the used mouse cultures even though an inhibitory effect is very likely.

The anti-proliferative effects on astrocytes might be worthwhile to be further explored, since gliosis was observed in PD patients and might sustain the progressive degeneration of dopaminergic neurons (Jenner and Olanow 2006; Renkawek et al. 1999). Furthermore, a potential efficiency of 9-me-BC treatment in glioblastoma therapy has to be investigated to show if the observed anti-proliferative effects on astrocytes might influence also the proliferation rate of degenerated cells. Actually, Cao et al. (2004) showed antitumor activities for numerous BC derivatives. 9-me-BC seems also to interact with the regulation of protein homeostasis since gene expression of Skp1 was increased. The major downstream target of PI3K is the kinase Akt, which seems to increase the number of dopaminergic neurons, their size and their sprouting into the striatum (Ries et al. 2009).

Dysfunctional protein degradation leads, e.g., to α-synuclein accumulation and aggregation over time in PD. A recent study showed that 9-me-BC decreases the protein levels of α-synuclein (Polanski et al. 2010). Probably, this effect might be explained by an improved protein homeostasis. In this context, metabolism of aggregated α-synuclein in astrocytes might be influenced and, as a result, a protection of dopaminergic neurons against debris of α-synuclein, since direct transfer of α-synuclein from neurons to astrocytes was reported (Lee et al. 2010). This theory is strengthened by the findings that 9-me-BC increased the gene expression of α-synuclein but decreased its protein content (Polanski et al. 2010), indicating a higher turnover rate of this protein.

Finally, 9-me-BC inhibited the activity of MAO-A and -B, which might contribute to the observed increased dopamine content, and anti-apoptotic properties in cell culture after 9-me-BC treatment (Hamann et al. 2008). Monoamine oxidase (MAO) is a mitochondrial bounded enzyme and exists in two isoforms: MAO-A, which deaminates preferentially serotonin, epinephrine, norepinephrine and melatonin; and MAO-B, which deaminates phenylethylamine, while dopamine is deaminated equally by both isoforms (Bortolato et al. 2008). There is evidence that elevated expression of astrocytic MAO-B leads to specific degeneration of dopaminergic neurons (Mallajosyula et al. 2008) while inhibition of MAO-A and -B exerted anti-apoptotic properties (Malorni et al. 1998; Maruyama et al. 2001; Szende et al. 2001). Actually, inhibitors of MAO-B are preferentially used in PD, since this isoform is predominant in the basal ganglia (Collins and Youdim 1970; Youdim and Holman 1975) and prefers to metabolize dopamine. However, MAO-A is present in dopaminergic neurons (Westlund et al. 1993) and ensures a physiologically lower level of dopamine (Youdim and Bakhle 2006). The PD-causing substance MPTP is oxidized by MAO-B into the direct acting neurotoxin MPP + which injures dopaminergic neurons by blocking the complex I of the respiratory chain. Recent findings confirmed the inhibition of MAO-B by 9-me-BC and additionally showed that 9-me-BC inhibits the oxidation of MPTP into MPP + and, as a result, show neuroprotective effects (Herraiz and Guillen 2011). Further, the MAO-A inhibitory properties of 9-me-BC might be of advantage in depression, which also occurs in advanced stage of PD (Blonder and Slevin 2011; Cummings 1992), since MAO-A inhibitors are already therapeutically used. MAO-mediated reactions produce neurotoxic substances like hydrogen peroxide, ammonia and aldehydes as byproducts. Probably, the neuroprotective effects of 9-me-BC might also contribute to the inhibition of MAO and thus to the decrease of these chemical species. Thereby, 9-me-BC showed IC50 values of 1 µM for MAO-A inhibition and of 15.5 µM for MAO-B. In comparison, rasagiline, the therapeutically used MAO-B inhibitor in PD, exerted IC50 values of 412 nM for MAO-A and of 4.43 nM for MAO-B (Youdim et al. 2001). The inhibition of both isoforms might explain the observed increased dopamine content, dopamine uptake and anti-apoptotic properties in cell culture after 9-me-BC treatment (Hamann et al. 2008).

In summary, 9-me-BC seems to be a reasonable candidate for disease-modifying PD treatment since its multimodal properties interfere with the multifactorial pathomechanisms of PD. Moreover, 9-me-BC is the only BC that showed neuroprotective and even neuroregenerative effects in in vitro (Hamann et al. 2008; Polanski et al. 2010) and in vivo (Wernicke et al. 2010) studies. However, further in vivo studies have to show the tolerance of 9-me-BC with possible side effects and reveal whether 9-me-BC merits further development as an investigational new drug.

## Electronic supplementary material

Below is the link to the electronic supplementary material.

Supplementary file 1 Supplement figure: Staining of GFAP (green), F4/80 (red) and Hoechst (blue) revealed less than 1 % of non-astrocytic cells within the astrocytic cultures (200 × magnification) (TIF 4249 kb)

## Abbreviations

2,9-dime-BC +: 2,9-Dimethyl-β-carbolinium ion
9-me-BC: 9-Methyl-β-carboline
BC: β-Carboline
BrdU: Bromdesoxyuridine
D24: Disprocynium 24
DA: Dopamine
DAT: Dopamine transporter
DDC: Dopa decarboxylase
DIV: Day in vitro
MAO: Monoamine oxidase
MPTP: 1-Methyl-4-phenyl-1,2,3,6-tetrahydropyridine
OCT: Organic cation transporter
PD: Parkinson’s disease
PI3K: Phosphatidylinositol 3-kinase
SN: Substantia nigra
TH: Tyrosine hydroxylase
