# Correction to: 9-Methyl-β-carboline inhibits monoamine oxidase activity and stimulates the expression of neurotrophic factors by astrocytes

**DOI:** 10.1007/s00702-021-02433-w

**Published:** 2021-11-15

**Authors:** Sebastian Keller, Witold Henryk Polanski, Christoph Enzensperger, Heinz Reichmann, Andreas Hermann, Gabriele Gille

**Affiliations:** 1 Department of Neurology, Technische Universität Dresden, Fetscherstr. 74, 01307 Dresden, Germany; 2 Department of Neurosurgery, Technische Universität Dresden, Fetscherstr. 74, 01307 Dresden, Germany; 3 Institute of Pharmacy, Friedrich Schiller University of Jena, Philosophenweg 14, 07743 Jena, Germany; 4 SmartDyeLivery GmbH, Botzstraße 5, 07743 Jena, Germany; 5 Translational Neurodegeneration Section “Albrecht-Kossel”, Department of Neurology and Center for Transdisciplinary Neurosciences Rostock (CTNR), University Medical Center Rostock, University of Rostock, 18147 Rostock, Germany; 6 German Center for Neurodegenerative Diseases (DZNE) Rostock/Greifswald, 18147 Rostock, Germany

## Correction to: Journal of Neural Transmission (2020) 127:999–1012 10.1007/s00702-020-02189-9

The article 9‑Methyl‑β‑carboline inhibits monoamine oxidase activity and stimulates the expression of neurotrophic factors by astrocytes, written by Sebastian Keller, Witold Henryk Polanski, Christoph Enzensperger, Heinz Reichmann, Andreas Hermann and Gabriele Gille, was originally published Online First without Open Access. After publication in volume 127, issue 7, pages 999–1012 the author decided to opt for Open Choice and to make the article an Open Access publication. Therefore, the copyright of the article has been changed to © The Author(s) 2021 and this article is licensed under a Creative Commons Attribution 4.0 International License, which permits use, sharing, adaptation, distribution and reproduction in any medium or format, as long as you give appropriate credit to the original author(s) and the source, provide a link to the Creative Commons licence, and indicate if changes were made. The images or other third party material in this article are included in the article's Creative Commons licence, unless indicated otherwise in a credit line to the material. If material is not included in the article's Creative Commons licence and your intended use is not permitted by statutory regulation or exceeds the permitted use, you will need to obtain permission directly from the copyright holder. To view a copy of this licence, visit http://creativecommons.org/licenses/by/4.0/.

Open Access funding enabled and organized by Projekt DEAL.

The original article has been corrected.

