# Supplementary figures and images for: 9-Methyl-β-carboline inhibits monoamine oxidase activity and stimulates the expression of neurotrophic factors by astrocytes
Source: J Neural Transm (Vienna). 2020 Apr 13;127(7):999–1012. doi: 10.1007/s00702-020-02189-9 (PMC8592951; doi:10.1007/s00702-020-02189-9)

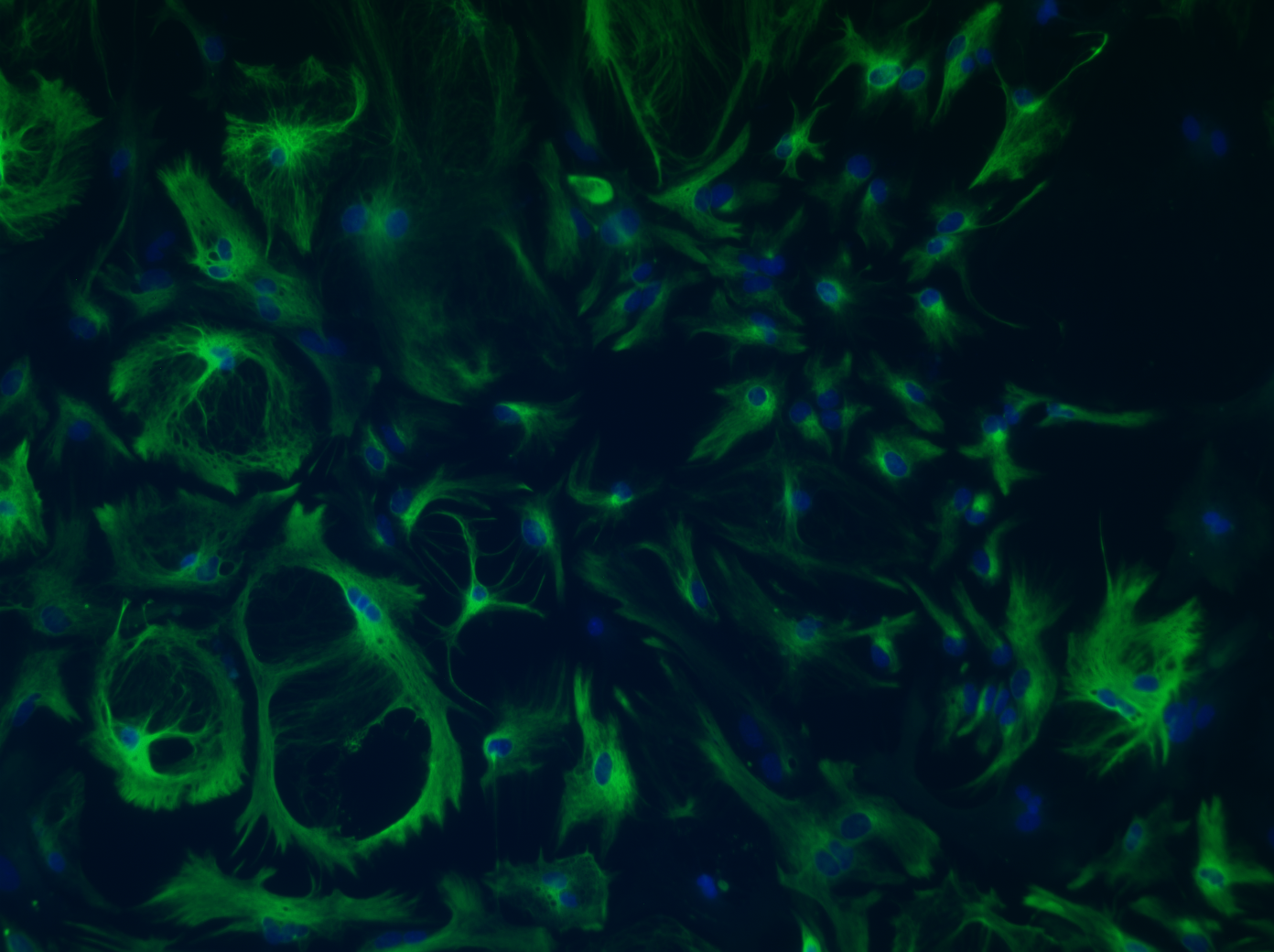

Supplement: Supplementary file 1 — Supplement figure: Staining of GFAP (green), F4/80 (red) and Hoechst (blue) revealed less than 1 % of non-astrocytic cells within the astrocytic cultures (200 × magnification) (TIF 4249 kb) [file 702_2020_2189_MOESM1_ESM.tif]
